# Antioxidant and anti‐inflammatory effects of Peanut (*Arachishypogaea L*.) skin extracts of various cultivars in oxidative‐damaged HepG2 cells and LPS‐induced raw 264.7 macrophages

**DOI:** 10.1002/fsn3.2064

**Published:** 2020-12-20

**Authors:** Min Young Kim, Hyun‐Joo Kim, Yu‐Young Lee, Mi Hyang Kim, Jin Young Lee, Mun Suk Kang, Bon Cheol Koo, Byong Won Lee

**Affiliations:** ^1^ Department of Central Area Crop Science National Institute of Crop Science Rural Development Administration Suwon Korea

**Keywords:** anti‐inflammatory effect, antioxidant effects, cultivar, peanut skin, polyphenols

## Abstract

This study was performed to investigate the distribution of phenolic compounds in the peanut skins of various cultivars, as well as their antioxidant and anti‐inflammatory effect *(Arachishypogaea* L. cv. K‐Ol, cv. Sinpalkwang, cv. Daan, cv. Heuksaeng) and extraction solvent. The major components of red peanut cultivars (K‐Ol, Sinpalkwang, and Daan) were identified as proanthocyanidin, catechin, gallic acid, coumaric acid, and hesperidine, whereas the major components of black peanut cultivar (Heuksaeng) were identified as anthocyanin, ferulic acid, and quercetin. The DPPH and ABTS radical scavenging activities, and FRAP values were the highest in Daan followed by Sinpalkwng, K‐Ol, and Heuksang. Furthermore, the skin extracts of red peanuts effectively improved cell viability, reactive oxygen species generation, MDA concentration, and antioxidant enzyme activity (GR, GPx, CAT, and superoxide dismutase) in oxidative stress‐induced HepG2 cells, and reduced the expression of pro‐inflammatory factors (NO, TNF‐α, IL‐6, and IL‐1β) in LPS‐stimulated RAW 264.7 macrophages. These results suggest that red peanut skin extracts could effectively mediate physiological activity and provide valuable information for the use of peanut byproducts as functional food materials.

## Graphical abstract

This study was performed to investigate the distribution of phenolic compounds in the peanut skins of various cultivars, as well as their antioxidant and anti‐inflammatory effect (Arachishypogaea L. cv. K‐Ol, cv. Sinpalkwang, cv. Daan, cv. Heuksaeng) and extraction solvent. The skin extracts of red peanuts effectively improved cell viability, reactive oxygen species generation, MDA concentration, and antioxidant enzyme activity (GR, GPx, CAT, and superoxide dismutase) in oxidative stress‐induced HepG2 cells, and reduced the expression of pro‐inflammatory factors (NO, TNF‐α, IL‐6, and IL‐1β) in LPS‐stimulated RAW 264.7 macrophages.

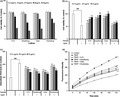

## 1 INTRODUCTION

An increase in the levels of cellular reactive oxygen species (ROS) above the levels tolerated by a cell's defenses results in oxidative stress (Ghosh & Myers, 1998). The high levels of ROS alter redox homeostasis and cause oxidative stress, which damages cellular proteins, lipids, and DNA. Oxidative stress has been recognized to be involved in the pathogenesis of aging and several degenerative diseases, such as cardiovascular and inflammatory diseases, diabetes, and cancer (Halliwell & Gutteridge, 1999). Furthermore, inflammatory response is a complex response to local injury or infection and involves various immune cells and numerous mediators (Kindt et al., 2007). While acute inflammation is essential for combating infection and tissue repair, excessive and uncontrolled inflammation is often associated with chronic diseases, such as metabolic disorders, atherosclerosis, and certain types of cancer (Chakrabarti et al., 2014). The role of food polyphenolics in the prevention of cardiovascular diseases and certain types of cancer is well recognized. Byproducts of seeds (Galali et al., 2020), such as the skins of brazil nut (John & Shahidi, 2010), almond (Wijeratne et al., 2006), and peanuts (Levy et al., 2017; Sarnoski et al., 2012) have been reported to be rich sources of polyphenolics and antioxidants.

Peanut (*Arachishypogaea L*.) is a critical oil crop that is widely cultivated in many countries, and it is an important food material that is consumed worldwide (Oldoni et al., 2016). Even though peanut skin is a small portion of the total seed, it was discarded 7.5 × 105 tons a year as a by‐product. But peanut skin contains several abundant polyphenolic compounds, such as flavonoids, phenolic acids, procyanidins, and anthocyanins, in approximately 90–150 mg/g of the dried skin (Nepote et al., 2010). The Rural Development Administration (RDA) of Korea conducted research on the development of peanut varieties with various functional characteristics such as Sinpalkwang (Pae, Hwang, Kim, et al., 2016), Daan (Pae, Lee, et al., 2017), K‐Ol (Pae, Hwang, Lee, et al., 2016), and Heuksaeng (Pae, Kim, et al., 2017). Sinpalkwang and Daan could be cultivated easily in high yields. Additionally, K‐Ol was found to comprise greater than 80% of oleic acid among the typical fatty acid composition, and Heuksaeng had a characteristic black color and high containing anthocyanin content. These developed peanut cultivars have different characteristics and phenolic compound profiles in peanut skin, whereas there is a lack of investigation on antioxidant and anti‐inflammatory effect in the HepG2 cell and RAW 264.7 macrophages.

Therefore, the objective of this study is to (a) investigate the composition of phenolic compounds in the skins of four peanut cultivars, as well as their antioxidant and anti‐inflammatory effects on tert‐butyl hydroperoxide (TBHP)‐induced oxidative stress in HepG2 cells and LPS‐stimulated RAW 264.7 macrophages and (b) evaluate the correlation between the phenolic compounds and the antioxidant and anti‐inflammatory characteristics to identify marker compounds in peanut skin.

## 2 MATERIALS AND METHODS

### 2.1 Sample preparation and extraction

Four types of peanut cultivars (*Arachishypogaea* L.) cv. Sinpalkwang, cv. K‐Ol, cv. Daan, and cv. Heuksaeng were used in this study. Two peanut cultivars, cv. Sinpalkwang and K‐Ol, were grown at the National Institute of Crop Science and Foundation of Agriculture Technology Commercialization & Transfer in Jeonju, South Korea, respectively, during the 2018 crop season. The other two peanut cultivars, cv. Daan and Heuksaeng, were grown at EM Food in Gochang, South Korea, during the 2018 crop season. The powdered samples (4 g) were extracted thrice with 80% ethanol (80 ml), 80% methanol (80 ml), 80% acetone (80 ml), and distilled water (80 ml) at room temperature for 1 hr using an ultrasonic bath. The extracts were then filtered and concentrated using a rotary evaporator under vacuum and were then freeze‐dried and stored at −20°C in an ultralow temperature freezer.

### 2.2 Determination of phenolic compounds

The total polyphenol and flavonoid levels were measured according to the method reported by Dewonto et al (Dewanto et al., 2002). The results are expressed as milligrams of gallic acid and catechin equivalents per gram of peanut skin (mg GAE/g peanut skin, mg CE/g peanut skin). Total proanthocyanidin levels were measured using the vanillin‐sulfuric acid method (Takahama et al., 2010). The results are expressed as milligrams of catechin equivalents per gram of the peanut skin (mg CE/g peanut skin). Total anthocyanin content was determined based on a reported pH differential method; these results were expressed in milligrams of cyaniding‐3‐glucose equivalents per gram of peanut skin (mg C3GE/g peanut skin) (Tchabo et al., 2015). The phenolic acid and anthocyanin composition of each extract was determined by high‐performance liquid chromatography (HPLC), as described by Kim et al. (2016) and Choung (2008), respectively.

### 2.3 Antioxidant activity

The radical scavenging activities of 1,1‐Diphenyl‐2‐picrylhydrazyl (DPPH) and 2,2‐azinobis (3‐ethyl benzothiazoline)‐6‐sulfonic acid (ABTS) were measured according to the method reported by Choi et al. (2006). Both radical scavenging activities were expressed as the Trolox‐equivalent antioxidant capacities (TEAC), as mg TE/g extract residue (ER). Also, the method of Benzie and Strain was used with modifications to measure the ferric‐reducing antioxidant power assay (Benzie & Strain, 1996); results are presented as mM Fe2 + equivalents.

### 2.4 Protective effect in oxidative‐damaged HepG2 cell

Human hepatoma HepG2 cells were obtained from the Korean Cell Line Bank (Seoul, Korea). The cell line was maintained in Dulbecco's modified Eagle's medium (DMEM), supplemented with 10% fetal bovine serum, 100 U/mL penicillin, and 50 μg/ml streptomycin at 37°C in an incubator with a 5% CO2 atmosphere. Human hepatoma HepG2 cell death was measured using a 3‐(4,5‐dimethylthiazol‐2‐yl)‐2,5‐diphenyltetrazoliumbromide (MTT) assay (Ishiyama et al., 1996). To determine cytoprotective effects against oxidative stress, HepG2 cells were seeded in a 96‐well plate at a density of 1.5 × 104 cells/well. After 24 hr, the culture medium (200 ml/well) was replaced with an FBS‐free medium (200 ml/well) containing various concentrations of extracts, after 12 hr, the culture medium containing the extract was discarded, and the cells were treated for 3 hr with the medium (200 ml/well) containing 200 mmol/L of TBHP to induce oxidative stress. We then evaluated the protective effect of extracts using the MTT assay. Intracellular ROS levels were quantified with a DCFH‐DA fluorescent probe, as previously described (Wang & Joseph, 1999). The fluorescence intensity, corresponding to the intracellular ROS generation, was measured with a fluorescence spectrophotometer (Perkin‐Elmer, Norwalk, CT, USA) for 2 hr at an excitation wavelength of 485 nm and an emission wavelength of 530 nm. To determine lipid peroxidation and antioxidant enzyme activity, the cells were harvested and lysed for 10 s using a Vibra‐Cell VCX 750 sonicator (Sonics&Materials, Inc., Newtown, CT, USA). The lysates were centrifuged at 10,000 × *g* for 10 min at 4°C, and the supernatants were used for protein and lipid peroxidation, and antioxidant enzyme assay according to the Ham et al (Ham et al., 2015).

### 2.5 Anti‐inflammation activity in LPS‐induced RAW 264.7 cell

RAW 264.7 macrophages were obtained from the Korean Cell Line Bank (*Seoul, Korea*). The cell line was maintained in DMEM, supplemented with 10% fetal bovine serum, 100 U/mL penicillin, and 50 μg/mL streptomycin at 37°C in an incubator with a 5% CO2 atmosphere. RAW 264.7 cell death was measured using a 3‐(4,5‐dimethylthiazol‐2‐yl)‐2,5‐diphenyltetrazoliumbromide (MTT) assay20. Cytotoxicity was calculated as a percentage of control cell viability. RAW 264.7 cells (5 × 104 cells/well) were seeded in 96‐well plates and incubated for 6 hr at 37°C. Cells were treated with or without LPS (0.5 μg/ml) and the indicated concentrations of soybean protein extract for 24 hr. Then, the concentration of NO, TNF‐α, IL‐6, and IL‐1β in the medium was measured using a Griess Reagent System (Promega, Madison, WI, USA) and Cymax TM Mouse cytokine ELISA assay kit (Ab PRONTIER, Seoul, Korea), respectively (Srisook et al., 2006).

### 2.6 Statistical analysis

All data are expressed as means ± standard deviations. Significant differences among treatments were determined by one‐way analysis of variance using Duncan's multiple range test, with SAS ver. 9.2 software (SAS Institute). The significance level was set to 0.05.

## 3 RESULTS AND DISCUSSION

### 3.1 Composition of phenolic compounds in peanut skin extracts

Plant phenolic compounds are currently among the most studied phytochemicals because of their biological functions, including antioxidant and anti‐inflammatory characteristics (Koleckar et al., 2008). The total polyphenol contents (TPC), total flavonoid contents (TFC), total proanthocyanidin contents (TPAC), and total anthocyanin contents (TAC) of the peanut skin according to cultivars and extraction solvents are shown in Table 1. Among the extraction solvents tested, 80% methanol and 80% acetone were found to be the most efficient solvent systems for extracting the polyphenolic compounds when compared with all other solvent systems used, and the levels of the TPCs ranged from 122.17 to 166.29 mg GAE/g peanut skin. The TFC results tended to be similar to TPC results. The recovery of the phenolic contents in different samples is influenced by the polarity of extraction solvents and the solubility of the particular compound in the solvent used for the extraction process (Sulaiman et al., 2011). When considering the effect of the cultivar on the TPC and TFC, the highest TPC was observed for the Daan cultivar in the case of the peanut skin extracted with 80% ethanol, 80% acetone, and distilled water. In contrast, the highest TPC, which was significantly higher than that of others, was observed for Heuksaeng (166.29 mg GAE/g peanut skin) and Daan (166.20 mg GAE/g peanut skin) in the case of the peanut skin extracted with 80% acetone and methanol, respectively. The high polyphenol content of the Heuksaeng cultivar was related to the anthocyanin content of the black pigment peanut cultivar. Also, these results are in close agreement with those reported by Larrauri et al. (2016) They observed that the extraction yield of the roasted and blanched peanut skin with 70% ethanol and distilled water ranged from 9.8% to 18.0%, and total the polyphenol contents of these extracts ranged from 295 to 672 mg GAE/g.

**TABLE 1:** Total polyphenol content (mg GAE/g), total flavonoid content (mg CE/g), total proanthocyanidin content (mg CE/g), and total anthocyanin contents (mg C3GE/g) of peanut skin extracts according to cultivars and extraction solvent

Cultivar	Solvent	TPC (mg GAE/g)	TFC (mg CE/g)	TPAC (mg CE/g)	TAC (mg C3GE/g)
K‐Ol	80% EtOH	104.90 ± 2.45^Db^	77.64 ± 2.49^Cc^	23.93 ± 0.27^Bb^	4.45 ± 0.13^Cc^
	80% MeOH	122.17 ± 3.98^Da^	83.38 ± 2.08^Cb^	30.03 ± 3.94^Ba^	7.43 ± 0.16^Cb^
	80% Aceton	125.79 ± 4.52^Da^	90.14 ± 3.02^Ca^	32.79 ± 1.99^Ca^	8.69 ± 0.37^Da^
	Water	58.11 ± 1.50^Cc^	40.48 ± 1.07^Cd^	10.65 ± 0.85^Cc^	1.47 ± 0.10^Cd^
Sinpalkwang	80% EtOH	112.22 ± 4.99^Cc^	84.16 ± 4.24^Bb^	25.71 ± 1.25^Bc^	4.48 ± 0.04^Cd^
	80% MeOH	131.83 ± 2.18^Cb^	98.42 ± 2.24^Ba^	34.02 ± 0.43^Bb^	7.85 ± 0.35^Cb^
	80% Aceton	139.97 ± 4.22^Ca^	101.17 ± 2.83^Ba^	43.66 ± 1.37^Ba^	9.56 ± 0.12^Ca^
	Water	52.73 ± 0.55^Dd^	33.25 ± 0.93^Dc^	13.03 ± 0.81^Bd^	1.52 ± 0.10^Cc^
Daan	80% EtOH	130.68 ± 0.42^Ac^	91.14 ± 1.11^Ac^	38.62 ± 1.15^Ab^	5.88 ± 0.17^Bb^
	80% MeOH	166.20 ± 2.13^Aa^	129.24 ± 2.32^Aa^	47.92 ± 0.51^Aa^	12.16 ± 0.21^Ba^
	80% Aceton	158.26 ± 0.74^Bb^	109.69 ± 2.43^Ab^	48.91 ± 0.57^Aa^	12.51 ± 0.41^Ba^
	Water	66.20 ± 2.15^Bd^	52.77 ± 0.42^Ad^	20.07 ± 0.58^Ac^	2.17 ± 0.11^Bb^
Heuksaeng	80% EtOH	123.65 ± 2.21^Bc^	49.90 ± 0.44^Dc^	8.68 ± 2.03^Cb^	13.85 ± 0.43^Ac^
	80% MeOH	149.21 ± 2.89^Bb^	63.48 ± 0.53^Db^	10.5 ± 1.95^Cab^	16.91 ± 0.12^Ab^
	80% Aceton	166.29 ± 1.34^Aa^	73.25 ± 0.67^Da^	13.09 ± 2.57^Da^	23.31 ± 0.45^Aa^
	Water	118.19 ± 2.45^Ad^	42.49 ± 0.68^Bd^	9.12 ± 1.20^Cb^	17.47 ± 0.29^Ab^

Note

Different capital letters and small letter in the same items indicate a significant difference (*p* < .05) among different cultivars and extraction solvent, respectively.

Abbreviations: TAC, Total anthocyanin content; TFC, Total flavonoid content; TPAC, Total proanthocyanidin content; TPC, Total polyphenol content.

**FIGURE 1:**
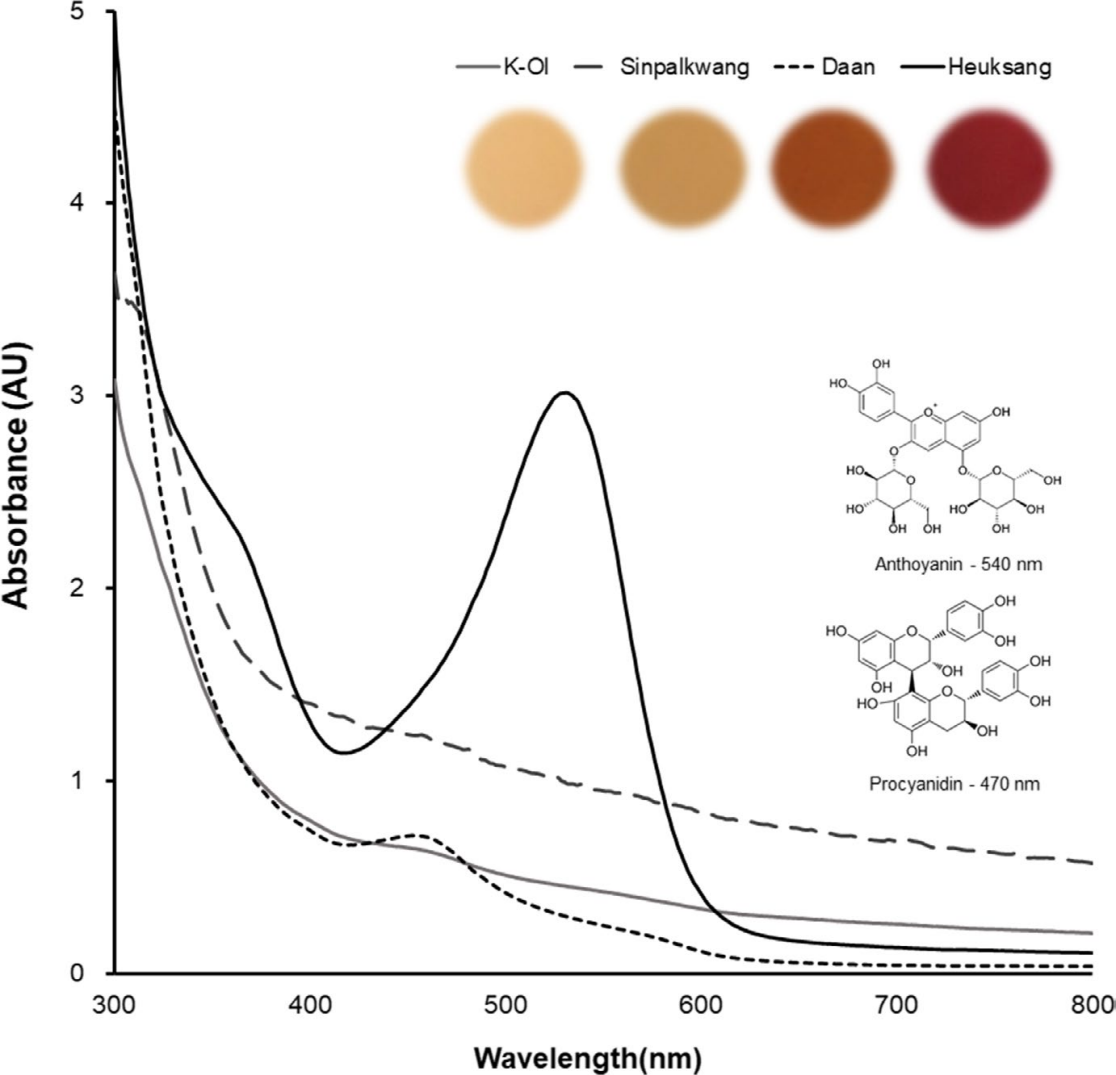
The wavelength scan (300–800 nm) of peanut skin extracts according to cultivars

Since the pigments of the peanut skin play an important role in physiological effects, there has been growing attention on the composition and medicinal use of peanut skin (Tatsuno et al., 2012). Therefore, we analyzed the pigment characteristics, such as UV absorption, total proanthocyanidin content (TPAC), and total anthocyanin content (TAC) of the peanut skin extracts of the various cultivars (Figure 1, Table 1). Peanut seeds have several skin colors, including red and black (Chukwumah et al., 2009). K‐Ol, Sinpalkwng, and Daan are red‐pigmented peanut cultivar, whereas Heuksaeng is a black‐pigmented peanut cultivar. These differences in the colors of the peanuts affected the UV spectra, TPAC, and TAC of the peanut skin extracts. As shown in Table 1, the total proanthoyanidin content was the highest in Daan (20.07–48.91 mg CE/g peanut skin) and was generally higher in the red peanut cultivars than those in the black ones. Meanwhile, the total anthocyanin contents were highest in Heuksang (13.85–23.31 mg C3GE/g peanut skin). Furthermore, the variations in the individual phenolics and anthocyanin profiles of the peanut skins according to their cultivar are shown in Table 2. Among the individual phenolics, the major compounds in the red peanut cultivars were catechin and ferulic acid, which accounted for approximately 85% of the total phenolics. However, in the case of the black peanut cultivar, ferulic acid was the major phenolics and accounted for approximately 75% of the total phenolics content. Moreover, anthocyanins, such as delpinidin‐3‐glucoside (1.88 mg/g), cyanidin‐3‐sorporoside (3.16 mg/g), and cyanidin‐3‐sambubitoside (1.26 mg/g), were only detected in black cultivars. Various studies have reported the phenolic compounds and anthocyanin according to the cultivar and skin color. Yu et al. (2005) reported that compounds found in red peanut skin are considered potent antioxidants, particularly, phenolic acid (ferulic acid, coumaric acid, and chlorogenic acid) and flavonoid (epicatechin, catechin, and Resveratrol). Also, Zhaoet al. (2017) found that the main anthocyanins were cyanidin‐3‐O‐sophoroside(C3So) and cyanidin‐3‐O‐sambubioside (C3Sa), which accounting forabout 90% of total anthocyanins in black peanut skin (BPS), and thereare also low content of Cyanidin‐3‐O‐glucosylrutinoside, Cyanidin‐3‐O‐xylosylrutinoside, etc. These differences in the peanut skin extracts depending on the color of the cultivar are believed to affect their antioxidant and anti‐inflammatory characteristics in the TBHP‐induced HepG2 cell and LPS‐stimulated RAW 264.7 macrophage tests.

**TABLE 2:** Phenolic acid contents (μg/g peanut skin) and anthocyanin contents (mg/g peanut skin) of peanut skins, according to their cultivar

	Cultivar			
	K‐Ol	Sinpalkwang	Daan	Heuksaeng
phenolics (μg/g)				
Catehin	845.87 ± 14.96^b^	931.93 ± 25.70^ab^	1,234.45 ± 64.12^a^	166.74 ± 7.90^c^
Gallic acid	55.30 ± 1.80^b^	65.42 ± 3.28^ab^	130.01 ± 14.53^a^	35.54 ± 2.25^c^
Coumaric acid	60.73 ± 0.50^b^	34.53 ± 1.56^c^	129.38 ± 9.69^a^	4.95 ± 0.11^d^
Ferulic acid	559.16 ± 15.59^c^	730.57 ± 19.15^b^	763.22 ± 127.38^b^	1,055.15 ± 13.20^a^
Hesperidin	8.82 ± 0.68^b^	8.99 ± 0.07^b^	12.75 ± 0.78^a^	2.74 ± 0.07^c^
Quercetin	60.30 ± 0.67^c^	36.68 ± 2.38^d^	94.55 ± 2.97^b^	132.91 ± 1.45^a^
Total	1,590.19 ± 1.08^c^	1,808.11 ± 28.47^b^	2,364.37 ± 159.57^a^	1,398.05 ± 15.17^d^
Anthocyanin (mg/g)				
Delpinidin‐3‐glucoside	ND	ND	ND	1.88 ± 0.36
Cyanidin‐3‐soporoside	ND	ND	ND	3.16 ± 0.37
Cyanidin‐3‐sambubioside	ND	ND	ND	1.26 ± 0.28
Cyanidin	ND	ND	0.17 ± 0.01	ND
Peonidin	ND	ND	0.06 ± 0.01	ND
Total	ND	ND	0.23 ± 0.00	6.3 ± 1.00

Note

Different small letters in the same item indicates a significant difference (*p* < .05) among different cultivars.

Abbreviation: ND, Not detected.

### 3.2 Effect of peanut skin extracts on radical scavenging activity and reducing power

**FIGURE 2:**
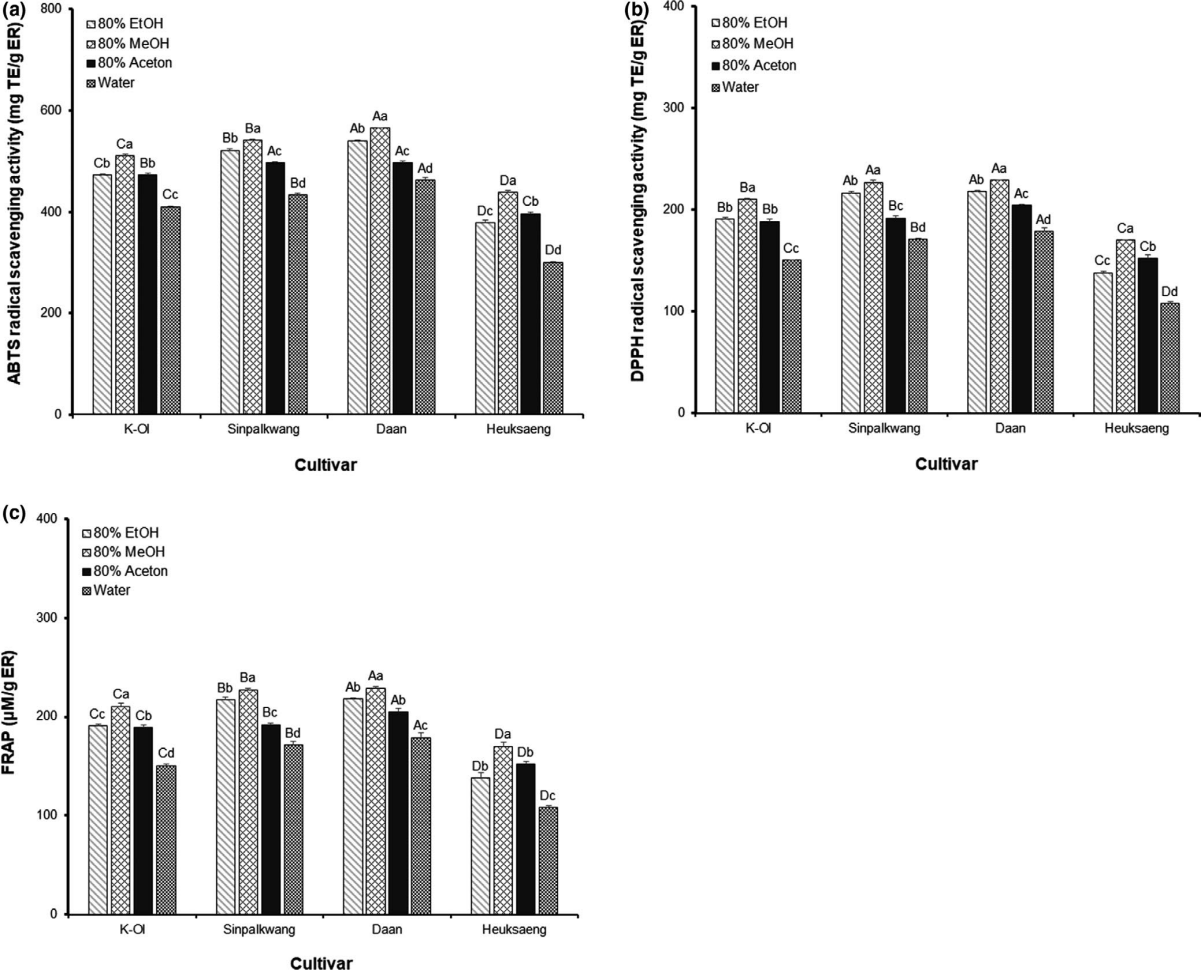
(a) ABTS (mg AAE/g extract), (b) DPPH (mg AAE/g extract) scavenging activity, and (c) FRAP (μM/g extract) of peanut skin extracts according to cultivars and extraction solvent. Different capital letters and small letter in the same items indicate a significant difference (*p* < .05) among different cultivars and extraction solvent, respectively

The effects of the cultivar and extraction solvents on the peanut skin extract's ABTS, DPPH radical scavenging activities, and reducing power (FRAP) are presented in Figure 2. The ABTS, DPPH radical scavenging activities, and FRAP values were highest in Daan (462.63–565.05 mg AAE/g extract, 178.80–228.92 mg AAE/g extract, and 278.38–451.29 μM/g extract), and were generally higher in the red peanut cultivars than the black peanut ones. Interestingly, the total polyphenol contents were not significantly different between Daan and Heuksang (Table 1), whereas the radical scavenging activity and reducing power was significantly higher in Daan than that in Heuksang (*p* < .05). These results are related to the difference in compositions of the phenolics that make up the total polyphenols. Muselík et al. (2007) reported in vitro antioxidant activity of isolated catechins, procyanidins, anthocyanins, and pyranoanthocyanins by using four methods that had different mechanisms. They concluded that procyanidins were among the in vitro tested groups and were the ones which showed higher antioxidant capacity, followed by catechins, anthocyanins, and pyranoanthocyanins. The antioxidant activity of the tested series of polyphenolic compounds correlated with the number of aromatic hydroxyl groups in the aqueous phase assays (Adhikari et al., 2019). In this study, we postulated that red peanut cultivars, containing a high amount of catechins and proanthocyanidins, which have a relatively large number of aromatic hydroxyl groups, showed higher antioxidant activity than the black peanut cultivar composed of anthocyanins.

### 3.3 Effect of peanut skin extract on endogenous antioxidant defense system and biomarker of oxidative stress in TBHP‐induced HepG2 cell

**FIGURE 3:**
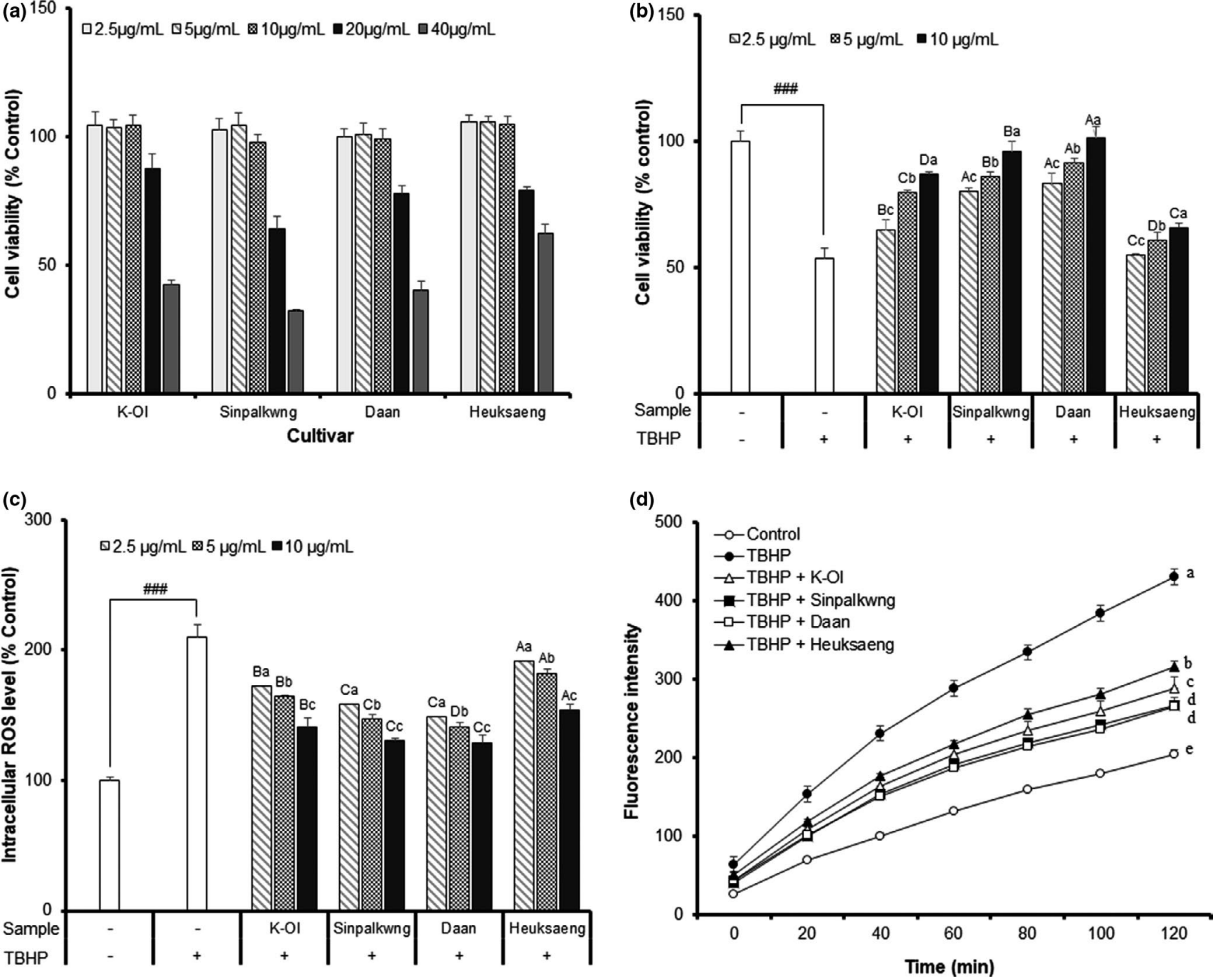
Effect of peanut skin extracts of different cultivars on (a) cell viability, (b) hepatoprotective effect, (c) intracellular ROS level, (d) fluorescence intensity for ROS in TBHP‐induced HepG2 cell. Different capital letters and small letter in the same items indicate a significant difference (*p* < .05) among different cultivars and extraction solvent, respectively

The hepatoprotective effects of the peanut skin extracts on oxidative stress‐induced HepG2 cell were investigated to determine protective effects, ROS inhibition, malondialdehyde (MDA) concentration, and antioxidant enzyme activity (glutathione reductase; GR, glutathione peroxidase; GPx, SOD, catalase; CAT). Before measuring the cytoprotective effects of the peanut skin extract on TBHP‐induced HepG2 cells, the cytotoxicity was estimated by MTT assay Figure 3a). Next, we evaluated the cytoprotective effects of the peanut skin extracts of the four different cultivars against TBHP‐induced oxidative damage (Figure 3b). These results demonstrate that the treatment with 500 μM TBHP significantly decreased cell viability by 53.68% compared to that in control cells. However, pretreatment of the peanut skin extract at 2.5–10 μg/ml significantly increased cell viability in a dose‐dependent manner, regardless of the identity of the cultivar. Especially, the highest protective effects from the TBHP‐induced stress in the HepG2 cells were observed in the peanut skin extracts of Daan and Sinpalkwng; these extracts increased cell viability to 101.19% and 95.97%, respectively, at a concentration of 10 μg/ml.

After evaluating the protective effects of the peanut skin extracts against oxidative stress, the levels of the ROS were analyzed in the TBHP‐induced HepG2 cells (Figure 3c,d). Accumulation of the cellular ROS is known to be a significant cause of intracellular damage and causes age‐related and metabolic diseases (Halliwell et al., 1992). Therefore, determination of the ROS released from the cell provides a good index of oxidative damage of the cell (Alía et al., 2006). As shown in Figure 3c,d, the TBHP treatment rapidly stimulated oxidative cellular stress and damage, elevating the release of ROS in HepG2 cell by 210.01% compared to the control cell without TBHP. However, the pretreatment of peanut skin extracts at 2.5–10 μg/ml significantly decreased the intracellular ROS level in the TBHP‐induced HepG2 cell in a dose‐dependent manner. These extracts of Daan and Sinpalkwng reduced the ROS level from 210.01% for the TBHP‐induced cellular oxidative stress to 130.18% and 129.19%, respectively, at a concentration of 10 μg/ml.

Next, the intracellular MDA concentration in the TBHP‐induced HepG2 cell protein was analyzed as a lipid peroxidation index (Suttnar et al., 2001). Additionally, the intracellular antioxidant enzymes play critical roles in the defense mechanism against oxidative damage (Alía et al., 2006). Oxidative stress (TBHP, 1 mM) treatment in HepG2 cells stimulated a significant increase of MDA concentration and the enzyme activities of CAT, SOD, GR, and GPx (Table 3). However, these increases were reversed by pretreatment with the peanut skin extracts at 10 μg/ml. Among them, the peanut skin extracts of Daan and Sinpalkwang cultivars were the most effective in reversing the increase in the MDA concentration and activities of CAT, SOD, GR, and GPx. Martín et al. (2010) reported that the pretreatment of epicatechin decreased the activity of the antioxidant enzymes against the TBHP‐induced oxidative damage.

**TABLE 3:** Effect of peanut skin extracts of cultivars on lipid peroxidation and antioxidant enzyme activities in HepG2 cells

Sample	^1^MDA	^2^GR	^3^GPx	^4^CAT	^5^SOD
Control	0.37 ± 0.05	4.78 ± 0.24	28.22 ± 1.09	11.49 ± 1.19	2.7 ± 0.13
TBHP	0.80 ± 0.03^***^	13.39 ± 0.79^***^	78.59 ± 3.63^***^	29.75 ± 1.67^***^	10.26 ± 0.40^***^
TBHP + KME 10	0.58 ± 0.02^b^	9.25 ± 1.16^a^	30.98 ± 1.50^b^	15.86 ± 0.60^b^	3.93 ± 0.09^b^
TBHP + SME 10	0.49 ± 0.01^c^	6.39 ± 0.66^b^	25.48 ± 1.06^c^	10.74 ± 0.35^c^	2.98 ± 0.05^d^
TBHP + DME 10	0.45 ± 0.02^d^	6.13 ± 0.39^b^	20.19 ± 1.07^d^	9.41 ± 0.46^d^	3.21 ± 0.02^c^
TBHP + HME 10	0.63 ± 0.02^a^	10.49 ± 0.33^a^	38.79 ± 1.43^a^	19.51 ± 0.90^a^	4.86 ± 0.16^a^

Note

Lipid peroxidation of ^1^malondialdehyde (MDA, nmol mg protein) and antioxidant enzyme activity of ^2^glutathione reductase (GR, μmol min mg^−1^ protein), ^3^glutathione peroxidase (GPx, μmol min mg^−1^ protein), ^4^catalase (CAT, μmol min mg^−1^ protein), and ^5^SOD, unit mg^‐1^ protein) were evaluated in HepG2 cells treated for 6 hr with the samples, followed by treatment for 4 hr with 500 μM TBHP. Values are means ± *SD* (*n* = 3).

Significant at ***p < .001

Various studies have reported the antioxidant properties of peanut skin phenolics, mainly due to their radical scavenging ability (Bodoira et al., 2017). However, no study has sought to identify the effect of peanut skin extracts on bioprotective effects against oxidative stress in the HepG2 cells. Hepatoprotective effects of proanthocyanidin and catechin from various plants against oxidative stress have been investigated in many studies. These protective effects against oxidative stress are in consistent with the other in vitro studies carried out with various cell types (Praphasawat et al., 2011). Pretreatment of the HepG2 cells with the oligomeric procyanidin fraction could reduce the decrease in cell viability induced by TBHP by reducing ROS generation and malondialdehyde formation (Kim et al., 2013). Furthermore, it has been reported that the MDA levels were reduced by various plant polyphenols such as catechin, epicatechin, proanthocyanidin, following oxidative stress. It has also been reported that flavonoids have the ability to interact with bio‐membranes and protect them from free radicals38, and that procyanidins trigger the upregulation of a battery of antioxidant and detoxification enzymes, which boost cellular defenses against oxidative stress (Saija et al., 1995). From the results of this study, we postulate that the red peanut cultivars, which contain high amounts of catechins and proanthocyanidins, possess the ability to impart strong protection against oxidative damage in HepG2 cells, by modulating ROS production, MDA generation, and antioxidant enzyme activities.

### 3.4 Effect of peanut skin extracts on nitric oxide (NO) production and pro‐inflammatory cytokine secretion in LPS‐stimulated RAW 264.7 macrophages

**FIGURE 4:**
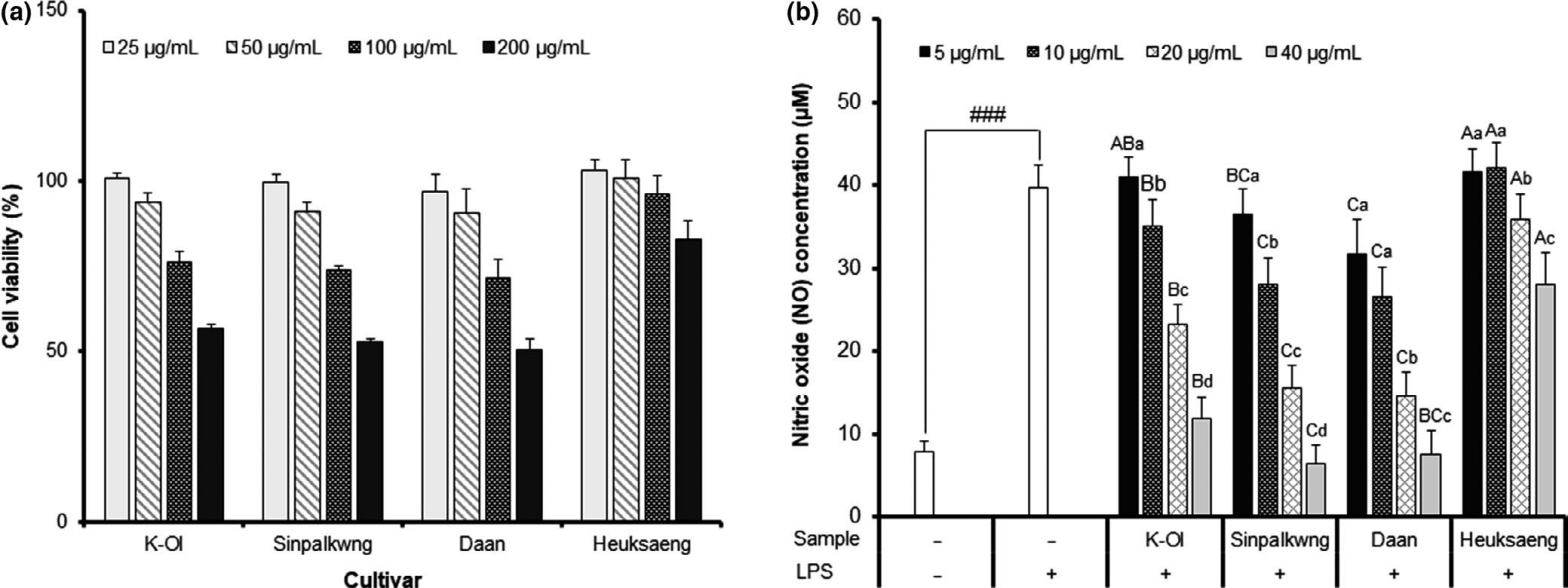
Effect peanut skin extracts of different cultivars on (a) Cell viability, (b) Nitric oxide concentration in LPS‐stimulated RAW 264.7 macrophages. Different capital letters and small letter in the same items indicate a significant difference (*p* < .05) among different cultivars and extraction solvent, respectively

The anti‐inflammatory effect of the peanut skin extracts was evaluated by measuring the levels of nitric oxide (NO), and the secretion of the pro‐inflammatory cytokines, such as TNF‐α, IL‐6, and IL‐1β. Before evaluating the anti‐inflammatory activities of the peanut skin extracts on the LPS‐stimulated RAW 264.7 cells, the cytotoxicity was estimated by MTT assay (Figure 3a). The effects of peanut skin extracts on the inhibition of NO production are presented in Figure 3b. NO production following LPS‐treatment for 24 hr (39.74 μM) significantly increased (*p* < .001) compared with that noted in the unstimulated RAW 264.7 cells (7.93 μM). However, the peanut skin extract (40 μg/ml) reduced the NO level by 70, 84, 81, and 30%, compared to the LPS‐stimulated controls of K‐Ol, Sinpalkwng, Daan, and Heuksang cultivar, respectively. Furthermore, TNF‐α, IL‐6, and IL‐1β are important pro‐inflammatory cytokines that are linked to the pathogenesis of many infectious and inflammatory diseases, including cancer (Chakrabarti et al., 2014). As shown in Figure 3c, LPS‐stimulated RAW 264.7 cells markedly up‐regulated TNF‐α, IL‐6, and IL‐1β production. The concentrations of TNF‐α, IL‐6, and IL‐1β in the media of the untreated cells (21.46, 1.46 ng/ml, not detected) were significantly increased by 446.85, 72.83, and 1.99 ng/ml after LPS stimulation, respectively. However, the peanut skin extract (40 μg/ml) decreased the TNF‐α, IL‐6, and IL‐1β levels by 19%–41%, 35%–88%, and 11%–38%, respectively, compared to the LPS‐stimulated control. Especially, peanut skin extracts of the Daan cultivar were most effective in reducing the concentration of TNF‐α, IL‐6, and IL‐1β, and these levels decreased by 264.15, 8.46, and 1.23 ng/ml, respectively. As a protective effect against HepG2 cells, the most effective inhibition of the major inflammatory factor (NO, TNF‐α, IL‐6, and IL‐1β) on the LPS‐stimulated RAW 264.7 cells was observed from the peanut skin extracts of Daan and Sinpalkwng. Anti‐inflammatory effects of proanthocyanidin polymers isolated from various plants, including peanut skin, have been reported in many studies. Tatsuno et al. (2012) reported that the proanthocyanidin polymers isolated from peanut skin inhibit the LPS‐induced cytokine (TNF‐α, IL‐6) production, which is attributable to the components of lower condensation. Therefore, we also conclude that the red peanut cultivars, which contain high amounts of proanthocyanidins, effectively enhance the anti‐inflammatory activities by modulating the major inflammatory factors (NO, TNF‐α, IL‐6, and IL‐1β) in LPS‐stimulated RAW264.7 macrophages (Figure 4).

### 3.5 Correlation analysis

**FIGURE 5:**
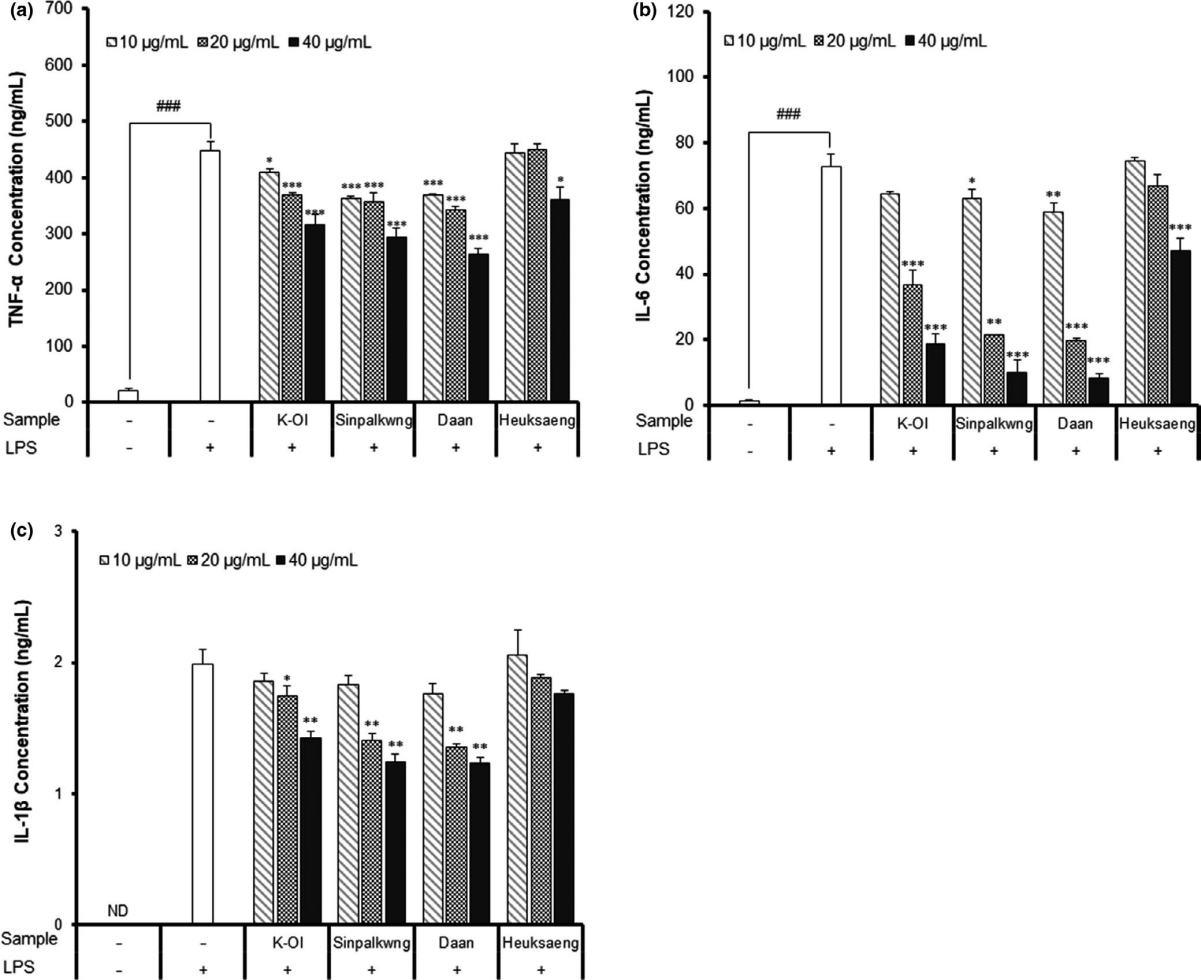
Effect of peanut skin extracts of different cultivars on (a) TNF‐α, (b)IL‐6, and (c) IL‐1β concentration in LPS‐stimulated RAW 264.7 macrophages. Different capital letters and small letter in the same items indicate a significant difference (*p* < .05) among different cultivars and extraction solvent, respectively

The correlation between the functional compounds and key factors on the antioxidant and anti‐inflammatory characteristics of peanut skin extracts was analyzed for various cultivars with different colors, and is expressed by the value of the correlation coefficient (*R*) presented in Table 4. In general, major functional compounds of the red peanut cultivars, such as flavonoids, proanthocyanidins, catechin, gallic acid, coumaric acid, and hesperidin, showed a significant positive correlation with radical scavenging activities, FRAP, and hepatoprotective effects. In contrast, they exhibited a negative correlation with biomarkers (MDA, GR, GPx, CAT, and SOD) for oxidative damage in HepG2 cells and inflammatory factors (NO, TNF‐α, IL‐6, IL‐1β) for LPS‐stimulated RAW 264.7 macrophages. In other words, flavonoids, proanthocyanidins, catechin, gallic acid, coumaric acid, and hesperidin exhibited relatively higher positive correlations with antioxidant and anti‐inflammatory capacities compared to TPC and anthocyanins. These results suggested that the functional compounds (proanthocyanidin, catechin, and so on) of red peanuts are more effective in protecting HepG2 cells against oxidative damage and the control of inflammation factors in RAW 264.7 macrophages than the functional compounds (anthocyanin) of black peanuts (Figure 5).

**TABLE 4:** Correlation analysis of biological activity and functional compounds in peanut skins

Functional compound	Antioxidant activity			Protective effect on TBHP‐induced HepG2							Anti‐inflammatory effect on LPS‐induced RAW 264.7			
	ABTS	DPPH	FRAP	Protective effect	ROS	MDA	GR	GPx	CAT	SOD	NO	TNF‐α	IL‐6	IL‐1β
Total polyphenol	−0.266	−0.389	0.034	−0.050	0.137	−0.018	0.067	0.088	0.069	0.287	0.444	0.179	0.413	0.340
Total flavonoid	0.830^**^	0.747^**^	0.933^**^	0.913^**^	−0.811^**^	−0.924^**^	−0.850^**^	−0.899^**^	−0.905^**^	−0.765^**^	−0.656^**^	−0.822^**^	−0.704^*^	−0.761^**^
Total proanthocyanidin	0.977^**^	0.954^**^	0.926^**^	0.897^**^	−0.847^**^	−0.884^**^	−0.831^**^	−0.98^**^	−0.939^**^	−0.913^**^	−0.924^**^	−0.942^**^	−0.942^**^	−0.940^**^
Total anthocyanin	−0.809^**^	−0.880^**^	−0.584^*^	−0.655^*^	0.676^*^	0.573	0.606^*^	0.691^*^	0.677^*^	0.805^**^	0.890^**^	0.742^**^	0.891^**^	0.846^**^
Phenolics														
Catehin	0.983^**^	0.965^**^	0.904^**^	0.907^**^	−0.827^**^	−0.890^**^	−0.837^**^	−0.952^**^	−0.910^**^	−0.893^**^	−0.920^**^	−0.933^**^	−0.954^**^	−0.953^**^
Gallic acid	0.812^**^	0.715^**^	0.891^**^	0.829^**^	−0.707^*^	−0.870^**^	−0.756^**^	−0.869^**^	−0.820^**^	−0.661^*^	−0.641^*^	−0.804^**^	−0.695^*^	−0.714^**^
Coumaric acid	0.792^**^	0.702^*^	0.815^**^	0.711^**^	−0.542	−0.754^**^	−0.615^*^	−0.828^**^	−0.712^**^	−0.580^*^	−0.626^*^	−0.735^**^	−0.694^*^	−0.691^*^
Ferulic acid	−0.622^*^	−0.692^*^	−0.358	−0.423	0.478	0.343	0.384	0.491	0.452	0.569	0.726^**^	0.571	0.722^**^	0.636^*^
Hesperidin	0.964^**^	0.930^**^	0.902^**^	0.864^**^	−0.763^**^	−0.864^**^	−0.818^**^	−0.947^**^	−0.885^**^	−0.843^**^	−0.864^**^	−0.911^**^	−0.918^**^	−0.913^**^
Quercetin	−0.632^*^	−0.747^**^	−0.402	−0.534	0.626^*^	0.423	0.523	0.501	0.555	0.753^**^	0.784^**^	0.589^*^	0.752^**^	0.727^**^
Anthocyanin														
Delphinidin‐3‐glucoside	−0.912^**^	−0.951^**^	−0.735^**^	−0.777^**^	0.762^**^	0.717^**^	0.720^**^	0.822^**^	0.795^**^	0.871^**^	0.934^**^	0.843^**^	0.954^**^	0.920^**^
Cyanidin‐3‐sorporoside	−0.912^**^	−0.950^**^	−0.735^**^	−0.776^**^	0.761^**^	0.717^**^	0.721^**^	0.819^**^	0.793^**^	0.869^**^	0.930^**^	0.840^**^	0.956^**^	0.919^**^
Cyanidin‐3‐sambubioside	−0.912^**^	−0.950^**^	−0.735^**^	−0.776^**^	0.760^**^	0.717^**^	0.721^**^	0.818^**^	0.792^**^	0.868^**^	0.927^**^	0.839^**^	0.956^**^	0.919^**^

Note

Significant at **p < .01, *p < .05.

## 4 CONCLUSION

This study was investigated the composition and distribution of phenolic compounds of peanut cultivars (Sinpalkwang, K‐Ol, Daan, Heuksaeng.), as well as the antioxidant and anti‐inflammatory effects of the skins of the four peanut cultivars. The total polyphenol contents were not significantly different between the Daan of red peanut cultivars and Heukasang of the black cultivar. In contrast, there is difference in the content of individual phenolics that made up the total polyphenol content. The total flavonoid content, total proanthocyanidin content, catechin, gallic acid, coumaric acid, and hesperidin were generally higher in red peanut cultivars (Sinpalkwng, K‐Ol, Daan) than in black peanut cultivars (Heukasang). Meanwhile, the anthocyanidin, ferulic acid, and quercetin contents were generally higher in black peanut cultivars than in red peanut cultivars. The ABTS and DPPH radical scavenging activities, and FRAP values were the highest in Daan, followed by Sinpalkwng, K‐Ol, and Heuksang, and were generally higher in red peanut cultivars than in black ones. In addition, our results show that the skin extracts of red peanut modulated cell viability, ROS generation, MDA concentration, and antioxidant enzyme activity (GR, GPx, CAT, and SOD) in oxidative stress‐induced HepG2 cells, and inhibited pro‐inflammatory factors (NO, TNF‐α, IL‐6, and IL‐1β) in LPS‐stimulated RAW 264.7 cells. Therefore, this study provides valuable information on the application of functional materials for improving the utilization of phenolic compounds extracted from peanut skins, depending on the different cultivars, as effective physiological agents.

## 6 CONFLICT OF INTEREST

The authors declare no conflict of interest.

## 7 ETHICAL APPROVAL

Neither animal nor human testing was involved in this study.
